# Study protocol—investigation of the Delirium Observation Screening Scale (DOSS) for the routine detection of delirium in the care home setting: a prospective cohort study

**DOI:** 10.1136/bmjopen-2015-009615

**Published:** 2016-06-20

**Authors:** Elizabeth Teale, John Young, Najma Siddiqi, Theresa Munyombwe, Jennifer Harrison, Marieke Schuurmanns

**Affiliations:** 1 Academic Unit of Elderly Care and Rehabilitation, Leeds University, Bradford, UK; 2 Academic Unit of Psychiatry and Behavioural Sciences, Leeds University and Bradford District Care NHS Foundation Trust, Leeds, UK; 3 Leeds Institute of Cardiovascular and Metabolic Medicine, Leeds University, Leeds, UK; 4 Centre for Cognitive Ageing and Cognitive Epidemiology and the Alzheimer Scotland Dementia Research Centre, University of Edinburgh, Edinburgh, UK; 5 Department of Rehabilitation, University Medical Center, Utrecht, Netherlands

**Keywords:** GERIATRIC MEDICINE, Delirium & cognitive disorders < PSYCHIATRY, Care Homes, Diagnosis

## Abstract

**Introduction:**

Delirium is a common and distressing condition associated with frailty, dementia and comorbidity. These are common in long-term care settings. Residents in care homes are therefore at particular risk of delirium. Despite this, methods to detect delirium in care homes are lacking, with existing diagnostic tools taking too long, or requiring specific training to deliver. This limits their feasibility for use for the routine detection of delirium by care home staff. Routine screening for delirium in care homes would allow timely attention to exacerbating factors to attenuate the episode, and facilitate future research into delirium in the care home environment.

**Methods:**

Residents from 4 large care homes will be asked to consent (or their consultees asked to provide a declaration of agreement) to participate in the study. Care home staff will administer the 25-item Delirium Observation Screening Scale (DOSS)—a delirium screening tool based on observed behaviours—and this will be tested against the research standard Confusion Assessment Method (CAM) administered by trained research assistants performed two times per week for all participating residents.

**Analysis:**

Sensitivity, specificity, positive and negative predictive values, likelihood ratios and a diagnostic OR will be calculated for the detection of delirium with the 25-item DOSS. The feasibility of routine delirium screening and the scaling properties of the 25-item DOSS will also be explored.

**Ethics and Dissemination:**

For residents lacking capacity to participate, a consultee will be approached for a declaration of agreement for inclusion in the study. Results will be published in peer-reviewed journals and disseminated in written format to clinical commissioning groups, general practitioners and relevant third parties.

**Trial registration number:**

ISRCTN14608554.

## Introduction

Delirium is a common and serious condition characterised by a sudden onset of altered cognition and impairments of attention, thinking and alertness. These symptoms tend to fluctuate over days or hours and often manifest as changes in behaviour. Delirium is associated with increased risk of new or accelerated cognitive problems, functional decline and death.^1^ Individuals suffering from an episode of delirium often have an altered perception of their surroundings and frequently misinterpret their environment. This may result in anxiety or unusual behaviours which can be distressing and frightening for the patient, their family and their carers.^2^

There are many risk factors for delirium, for example: dementia, deafness, poor vision, dehydration, pain, constipation and medications. These risk factors are prevalent in care homes where residents may be frail with multiple comorbidities including dementia, further increasing the risk of developing delirium.^3^ A 2009 BUPA survey revealed that 44% of care home residents had a diagnosis of dementia, and 60% were classed as ‘frail elderly’ at the time of death.^4^ It is therefore anticipated that delirium should be a common problem in residents of care homes. In 2010, the National Institute for Health and Care Excellence (NICE) guidelines for the diagnosis, prevention and management of delirium highlighted the paucity of research literature detailing the incidence and prevalence of delirium in long-term care settings.^5^ This may be, in part, due to the significant challenges in detecting delirium, and conducting research in care homes. A simple, valid delirium screening tool that can be reliably administered by care home staff would improve delirium detection and might also have utility for future research into delirium in the care home setting.

There are currently no laboratory tests or biomarkers to establish the diagnosis. Detection of delirium relies on identifying sudden changes in an individual's behaviour from their baseline. Care home staff are particularly well placed to detect these changes. Existing diagnostic tools for detection of delirium operationalise the diagnostic criteria of the Diagnostic Manual for Mental Disorders, Fourth Edition (DSM-IV).^6^ These tools require time and training to administer which has limited their uptake into routine care.

For an instrument to have utility for delirium detection in routine care in the care home setting, it should be brief and not require specialist knowledge or training to administer. It should have high sensitivity (a low false-negative rate), and acceptable specificity (a high true-negative rate).

Fourteen reviews of delirium assessment instruments have identified six externally validated instruments that may be administered by nursing staff. Of these, two may have utility as screening tools for delirium in the care home environment. These are the Delirium Observation Screening Scale (DOSS)^7^ and the Nursing Delirium Screening Scale (NuDESC).^8^ Sensitivity of the DOSS is comparable with the NuDESC for detection of delirium (pooled sensitivity for the DOSS is 92% (95% CIs 74% to 98% 2 studies, 178 participants) vs 96% for the NuDESC (95% CI 80% to 100% 1 study, 100 participants)).^9^ However, the specificity of the DOSS is higher (82% (95% CI 66% to 92%) vs 69% (95% CI 59% to 79%)); therefore, the DOSS was chosen as the candidate instrument for the study. The DOSS comprises items which seek objective assessment of the behavioural aspects of the DSM-IV delirium diagnostic criteria: onset of a short period of altered consciousness; reduced ability to focus, sustain or shift attention; a change in cognition (memory deficit, disorientation, language disturbance); and perceptual disturbance not better accounted for by a pre-existing, established or evolving dementia. The DSM also stipulates that, for a diagnosis of delirium, there is evidence from the clinical assessment that these changes are associated with a general medical condition and that there is fluctuation during the course of the day.

The psychometric properties of the DOSS have been assessed in acute and rehabilitation settings,^7^ ^10^ but the utility of the DOSS in care homes has not been evaluated. This study aims to determine how the DOSS performs as a screening instrument for delirium when administered by care home staff in comparison to a research standard assessment based on the Confusion Assessment Method (CAM).^11^

## Methods and analysis

The DOSS study is a prospective observational cohort study, performed in UK long-term care settings over 24 months (May 2014 to May 2016). The 25-item DOSS, performed once daily by care home staff, will be measured against the temporally closest CAM (a research standard) performed two times per week by research staff for all participants. Feasibility of routine daily administration of the DOSS will be investigated by examining patterns and rates of missing data on care home staff completed DOSS assessments. To determine the diagnostic test accuracy of the DOSS, sensitivity, specificity, positive and negative predictive values and likelihood ratios of the DOSS for the detection of delirium will be calculated. The CAM has been validated as a measure of delirium severity^12^ and this will allow for a further investigation to determine whether the DOSS may also be used as a measure of delirium severity. Repeated daily DOSS measurements will be used to examine the scaling properties of the 25-item DOSS instrument. Item response theory will be used to determine whether the questions are measuring the same underlying constructs (delirium) and whether there are any redundant questions which may be removed to create a shorter, care home-specific DOSS.^13^

### Recruitment

#### Study sites

Up to four large independent provider care homes (∼100 residents each) providing residential, nursing or specialist dementia care for older people in Leeds, Bradford and York will be eligible as study sites. Managers of eligible care homes will be approached regarding participation in the study. Inclusion as a study site will be based on agreement from the care home manager to embed the daily measurement of the 25-item DOSS, and to release staff for training sessions in delirium and administration of the DOSS instrument. Data on size (numbers of residents and staff), type of care (residential, nursing or specialist dementia care) and admission rate per bed will be collected for each participating care home. Specialist care homes are excluded unless the specialty is dementia care. Care homes participating in other studies likely to impact on delirium incidence (eg, delirium prevention studies) are also excluded.

### Participants

Individuals resident in any of the care home study sites are eligible to participate in the study. Residents under 65 years are at lower risk of delirium^5^ and are therefore excluded from the study. Residents approaching end of life or in receipt of palliative care (as advised by care home staff), and those with severe communication difficulties (unable to complete the CAM) will be excluded.

### Consent procedures

The properties of the DOSS screening instrument are being tested in a population with a high prevalence of dementia. It is therefore likely that a significant number of potential participants will lack mental capacity to provide written informed consent to participate in the study. It is important that these individuals are not excluded from participating in the research to ensure that the study sample is representative and to maximise generalisability of the results to those potentially most likely to benefit from the study findings.

Residents eligible to participate in the study will be approached and asked to provide informed consent through a combined consent–capacity process. Where a resident lacks capacity to consent to participate in the study, a declaration for participation will be sought from a personal consultee in accordance with the Mental Capacity Act (2005). If no personal consultee is available, a member of care home staff will be asked to act as a nominated consultee, acting in the best interests of the resident and in accordance with any pre-expressed views or wishes. Capacity may fluctuate during the course of the study, due to the fluctuating course of delirium. We will ask potential participants whether they consent to continue with study assessments in the event that they subsequently lose capacity. Residents who have provided this consent will continue in the study unless there is a clear expressed wish from the resident, a family member or carer that they wish to withdraw.

### Training

#### Care home staff

Most care home staff will have experience of caring for residents with delirium but it may not have been identified as such. Specific training in delirium will be provided in interactive small group sessions drawing on the existing experience of care home staff in recognising behaviour change in residents with delirium, putting this into the context of the items of the 25-item DOSS. Written training manuals will be provided for care home staff describing delirium and delirium risk factors, the completion of the DOSS instrument and study procedures. Tests of DOSS inter-rater reliability will be performed monthly by care home staff.

#### Research staff

Research assistants will be trained in delirium assessment according to the methods described in the CAM administration manual.^11^ This training will include a scenario-based delirium detection session using the CAM (and the CAM-severity scoring methodology). CAM assessments will be performed according to the methodology outlined in the CAM administration manual.^11^ Researchers will be observed performing the CAM assessments in the care home environment by a consultant geriatrician and tests of CAM inter-rater reliability will be performed monthly.

### Study assessments

#### Baseline

The number of residents, type of care home (nursing or residential) and Care Quality Commission rating will be recorded for each participating care home.

Data collected for all potentially eligible participants will include age, sex, an existing dementia diagnosis or a positive response to the case finding question used for the UK National Commissioning for Quality and Innovation (CQUIN), ‘*has the person been more forgetful in the last 12 months to the extent that it has significantly affected their daily life?*’.^14^ For residents who are subsequently recruited into the study, a validated screening instrument for cognitive impairment comprising 10 questions, the Abbreviated Mental Test Score (AMTS), will be performed at baseline.^15^ A baseline CAM will also be performed.

#### Study assessments

Two delirium assessment instruments will be used throughout the 9 months of the study. These are the CAM performed two times per week by research staff and the 25-item DOSS performed once daily by care home staff, embedded into routine care. In contrast to the 13-item DOSS, the 25-item DOSS includes behaviours which span a full 24-hour period (ie, including night-time behaviours). If care home staff reflect back on a resident's behaviour over the course of a shift and use additional information obtained from care home records and handovers, it should be possible to identify whether there have been fluctuations in a resident's behaviour without the need to perform more than one DOSS assessment per day. Care home staff will be blinded to CAM assessments, and research assistants will be blinded to DOSS assessments, which will be stored separately from the care home record. Since symptom fluctuation is one of the diagnostic criteria for delirium, testing the sensitivity and specificity of the DOSS against the CAM relies on measurements being made close together such that variation is not falsely attributed to the instruments. The nearest date and timed DOSS measurement will be tested against the research assistant-administered CAM.

#### CAM assessments

The CAM is an operationalised approach to applying the DSM delirium criteria. It has been used extensively in research and, to a lesser extent, in routine care. Administration takes 5–10 min. The CAM is positive, and delirium present, if there is sudden onset and a fluctuating course of inattention and either disorganised thinking or an altered conscious level.^11^ A structured approach to the identification of inattention and disorganised thinking will be adopted as described by O'Regan *et al*.^16^ Inattention will be assessed formally through the ‘Months of the Year Backwards’ (MOTYB) test.^16^ Disorganised thinking will be assessed using the methods outlined in the CAM administration manual.^11^ In addition, questions aimed at testing abstract reasoning (eg, explaining the meaning of a common proverb) will be used.^16^ Scoring of the CAM is described in detail in the CAM handbook;^11^ the CAM-severity scoring system will be adopted to allow the severity of delirium to be graded.^12^ The CAM assessments will be entered electronically into encrypted laptop computers.

#### DOSS assessments

The DOSS identifies specific features of affect or behaviour to facilitate recognition of DSM defined criteria for delirium. The DOSS is based on non-technical observations from nurses or carers as they attend to patients during routine care. Only minimal training is required for administration. This study will examine the 25-item DOSS rather than the 13-item scale (which is more weighted towards hyperactive delirium). The hypoactive delirium subtype has been shown to be twice as common as hyperactive delirium in the postacute setting^17^ and is associated with poor outcomes.^18^ However, hypoactive delirium is poorly detected, particularly by nursing staff.^19^ Methods to improve the detection of hypoactive delirium by nursing staff in the care home setting are therefore desirable. The full 25-item DOSS can be completed in 5 min.^20^ The 25-item DOSS has been shown to have content validity and internal consistency (Cronbach's α 0.93 and 0.96, respectively).^7^ Concurrent validities against international standard delirium diagnostic and severity instruments (the CAM and the Delirium Rating Scale—Revised—98)^7^ ^10^ are good. In order to ensure that the 25-item DOSS is relevant to the UK care home environment, it has been necessary to reword some items (eg, to remove reference to interventions that would not be expected in the care home environment, eg, intravenous lines).

When the 13-item DOSS was derived from the 25-item DOSS, the original Likert scoring scale was dichotomised for each item to simply record whether a particular behaviour has, or has not, been observed during the course of a shift.^21^ For this study, each item of the 25-item DOSS will be scored 0 or 1 with a higher score indicating behaviour more in keeping with delirium (some items are reverse scored).

### Sample size calculation

Measurement of sensitivity of the DOSS requires identification of cases where CAM and DOSS are positive. Sample size calculation is therefore based on the number of positive delirium episodes (identified with the researcher-administered CAM) required to measure the sensitivity of the DOSS with 95% confidence that the observed value is within 95% of the true value. Using the pooled estimate for sensitivity (92%) of the DOSS from previous studies,^9^ and the normal approximation to the binomial proportion distribution, there would need to be 113 measurements of CAM positive delirium during the study period.

The study involves repeated assessments; there is therefore likely to be correlation between serial CAM measurements. An episode of delirium lasts, on average, 11 days in the long-term care setting.^22^ CAM measurements are 4 days apart. It is likely that there will be at least two CAM measurements during each episode of delirium. We have therefore assumed at least moderate correlation between CAM measurements (intraclass correlation coefficient of 0.5). Applying an inflation factor (of 1.5) to account for the study design effect due to the repeated measures would require 170 CAM positive measurements over the 9-month study period. A recent study of delirium in Canadian long-term care settings offers the best available estimate of delirium incidence in care homes of 21.8% in 24 weeks.^1^ We estimate that 170 CAM positive readings would be made in 258 residents over 36 weeks.

### Data analysis plan

We aim to evaluate the feasibility of embedding the existing DOSS instrument into routine care rather than to evaluate the content of the questionnaire. Feasibility of administration of the DOSS by care home staff will therefore be examined through exploring the rates of missing data, and by examining the intraclass correlation coefficients between staff-administered DOSS assessments.

A receiver operating curve will be plotted to determine the optimal cut-point for the 25-item DOSS. Sensitivity, specificity, positive and negative predictive values, likelihood ratios positive and negative, and a diagnostic OR will be calculated with 95% confidence limits.

Correlation of the 25-item DOSS with the CAM severity score will be used to determine whether the DOSS may be used as a marker of delirium severity.

Item response theory (non-parametric and parametric if appropriate) will be performed to determine the scaling properties of the 25-item DOSS and to identify whether there are any redundant items to form a care home-specific version of the DOSS.

## Ethics and dissemination

The aim of this study is to identify delirium in a population at high risk from the condition. It is likely that this will increase the diagnosis of delirium in care homes. This may increase the requirement for medical assessment and input (eg, from general practitioners). General practitioners will be informed in writing when one of their patients is recruited into the study. Where a diagnosis of delirium is made by the research assistant (with the CAM), the care home manager will be informed in writing on the same day, such that appropriate action may be taken according to local procedures.

Results of the study will be published in peer-reviewed scientific journals. Reports will be prepared for, and disseminated to, Leeds, Bradford and York Clinical Commissioning Groups (CCGs), local General Practitioners, Age UK and the British Geriatrics Society. Lay summaries will be sent to participant care homes for dissemination to staff, residents and relatives.
